# The feasibility and ongoing use of electronic decision support to strengthen the implementation of IMCI in KwaZulu-Natal, South Africa

**DOI:** 10.1186/s12887-022-03147-y

**Published:** 2022-02-07

**Authors:** Cecilie Jensen, Neil H. McKerrow

**Affiliations:** 1 Health Systems Strengthening Unit, Health Systems Trust, Durban, South Africa; 2 KwaZulu-Natal Department of Health, Paediatrics and Child Health, Pietermaritzburg, South Africa; 3 Department of Paediatrics and Child Health, University of Cape Town, Cape Town, South Africa; 4 Department of Paediatrics and Child Health, University of KwaZulu-Natal, Durban, South Africa

**Keywords:** Integrated Management of Childhood Illness (IMCI), Electronic health (eHealth), Clinical decision support systems (CDSS), Feasibility, Sustainability, Implementation, Health systems, Coronavirus 2019 (COVID-19)

## Abstract

**Background:**

Continued efforts are required to reduce preventable child deaths. User-friendly Integrated Management of Childhood Illness (IMCI) implementation tools and supervision systems are needed to strengthen the quality of child health services in South Africa. A 2018 pilot implementation of electronic IMCI case management algorithms in KwaZulu-Natal demonstrated good uptake and acceptance at primary care clinics. We aimed to investigate whether ongoing electronic IMCI implementation is feasible within the existing Department of Health infrastructure and resources.

**Methods:**

In a mixed methods descriptive study, the electronic IMCI (eIMCI) implementation was extended to 22 health facilities in uMgungundlovu district from November 2019 to February 2021. Training, mentoring, supervision and IT support were provided by a dedicated project team. Programme use was tracked, quarterly assessments of the service delivery platform were undertaken and in-depth interviews were conducted with facility managers.

**Results:**

From December 2019 – January 2021, 9 684 eIMCI records were completed across 20 facilities, with a median uptake of 29 records per clinic per month and a mean (range) proportion of child consultations using eIMCI of 15% (1–46%). The local COVID-19-related movement restrictions and epidemic peaks coincided with declines in the monthly eIMCI uptake. Substantial inter- and intra-facility variations in use were observed, with the use being positively associated with the allocation of an eIMCI trained nurse (*p* < 0.001) and the clinician workload (*p* = 0.032).

**Conclusion:**

The ongoing eIMCI uptake was sporadic and the implementation undermined by barriers such as low post-training deployment of nurses; poor capacity in the DoH for IT support; and COVID-19-related disruptions in service delivery. Scaling eIMCI in South Africa would rely on resolving these challenges.

## Introduction

Continued efforts are required to reduce preventable child deaths, particularly as the direct and indirect effects of the COVID-19 pandemic are threatening to reverse recent gains in child survival [1–5]. To decrease the under-5 mortality to < 25 per 1 000 live births by 2030, South Africa needs to maintain and strengthen programmes aimed at improving child survival [6]. Evidence-based strategies at the primary care level include the Integrated Management of Childhood Illness (IMCI) approach to improve healthcare worker skills, health systems and community practices. However, incomplete implementation and suboptimal case management has hampered the effectiveness of the IMCI strategy globally and locally [1, 2, 7–10]. Reported barriers are often mirrored across various low-and-middle income settings and frequently include time-consuming and expensive training; lack of post-training support for healthcare workers; supply-chain issues for drugs and essential equipment; and fragmentation of health systems with poor political and financial support for IMCI [1, 8–10]. Needs have been identified for user-friendly guidelines and tools and better mentoring and supervision of healthcare workers [1, 8, 10, 11]. As the IMCI case management process is algorithmic it is well suited to digitalization and automation. Using electronic IMCI algorithms (compared to conventional IMCI case management) has in various low- and middle-income settings been associated with improvements in several quality of care indicators, such as protocol adherence, nutrition screening, correct diagnosis, correct treatment administration, vitamin A and deworming supplementation and reduced unnecessary antibiotic prescription [12–15]. A 2018 pilot implementation of electronic IMCI (eIMCI) case management in KwaZulu-Natal demonstrated good uptake and acceptance among caregivers of sick children, nurses providing care and managers at primary care facilities. The uptake of eIMCI was most strongly related to the nurses’ computer literacy levels. Common themes in the maternal interviews included improved comprehensiveness, correctness and efficiency of service [16]. A phase 2 implementation has subsequently been carried out with scale-up to additional sites within the same health district. Although there are reports of good initial acceptance of electronic health records, electronic clinical decision support systems, and electronic monitoring and evaluation systems in the South African setting [17–19], evaluations assessing their sustained use and feasibility within real-life health systems are lacking.

This study aims to evaluate the feasibility of ongoing electronic IMCI implementation within the existing Department of Health infrastructure and resources.

## Methods

The electronic IMCI programme was implemented in 22 health facilities in uMgungundlovu district from November 2019 to February 2021. A mixed methods approach was employed to evaluate the feasibility of ongoing implementation in the local context. Primary study outcomes included: 1. The description of ongoing uptake of electronic IMCI; 2. The identification and quantification of key barriers to ongoing implementation.

### Programme implementation

The eIMCI software development has been described briefly in a previous acceptability evaluation [16]. From inception, the project was conducted in collaboration with the provincial Department of Health (DoH) with the premise of roll-out within existing DoH information technology (IT) infrastructure. The project team could therefore not provide computers, computer parts or internet connection where these were lacking, and facilities needed to wait for the relevant DoH processes to take place. The phase two implementation commenced in November 2019 and built on an initial 2018 roll-out in the same district. Following district engagement and site visits to assess the availability of computers, seven new facilities, from a possible 38, were added to the 15 pilot sites. The capacitation of implementing nurses included four-day training workshops with subsequent mentoring and ongoing support at the facilities. Training workshops were held for IMCI trained nurses from newly included sites as well as additional IMCI trained nurses from the pilot sites. The eIMCI training model had been tested and refined during the 2018 implementation. Day one of the training was dedicated to basic computer literacy skills. The second day covered an overview of the IMCI case management process and recent guideline updates in the areas of malnutrition, HIV, prevention of mother-to-child transmission (PMTCT) and tuberculosis. During training days three and four, participants completed sets of case scenarios using the eIMCI system on training computers with individual feedback from the facilitators. Role-plays were conducted to practice maintaining the patient and caregiver interaction whilst operating the computer. Additionally, case scenarios were completed with conventional IMCI forms so participants could compare the electronic to the paper-based case management process. Participants also underwent pre-training assessments in computer literacy and IMCI knowledge.

Following the training workshops, all participants received at least monthly mentoring and support from a dedicated project mentor for the duration of the study period, as well as on-demand assistance with technical challenges. An accreditation process was employed to encourage the participants to practice after the training and to ensure a good standard of implementation. Criteria for nurses to be certified as competent in eIMCI implementation included: 1. having attended a full four-day course, 2. having completed at least 40 child consultations using the eIMCI system at their facility, and 3. obtaining a score of at least 85% during three or more skills assessment (observed consultations) with the mentor. The mentor also worked with the nurses and facility managers to set daily targets for eIMCI use based on the facility caseload of children and the individual nurses’ computer literacy levels. Additionally, telephonic and on-site problem-solving and IT support was provided by the project team. The initial focus was on training and mentoring of eIMCI trained nurses with plans of a gradual transition to supervision, monitoring and handover to the Department of Health.

### Study procedures and participants

Principal study interventions included the expansion of eIMCI to additional eligible sites, documenting the ongoing use, identifying barriers to use and key informant interviews. Eligibility criteria for inclusion of health facilities in eIMCI implementation included primary care facilities, i.e. primary healthcare clinics (PHCs) and community health centres (CHCs), and access to a functional computer in the IMCI consultation room. Due to the limited availability of computers, the sampling frame in uMgungundlovu district was insufficient for randomization, however, representation of different settings (rural, urban and peri-urban) was ensured. Twenty-two healthcare facilities were included, of which 15 were pilot facilities and seven were new facilities. In the quarterly site assessments, participants represented healthcare facilities rather than individual healthcare workers. For the in-depth interviews of the facility managers, eight facilities were randomly selected from all sites.

### Data collection and analysis

Baseline data collection included site assessments capturing facility characteristics and the information technology (IT) infrastructure. A database was kept of eIMCI trained nurses documenting their work allocation, eIMCI use, skills assessment scores during mentoring, and dates of certification. The uptake of eIMCI was tracked throughout the implementation period by aggregating patient data files from the facilities. Automatic synchronization between the facilities and the central server was prevented by facility level internet challenges, which necessitated the manual extraction of data files from the computers during site visits. Data elements obtained electronically included the number of electronic child assessments completed per facility per day and week. Records were kept of weekly team meetings with the nurse mentor and technical support staff in which the progress with participant mentoring and IT challenges were discussed. The IT support team also logged computer problems reported by the sites.

Quarterly site assessments were conducted by a field worker (NN) and included the quantification of elements anticipated to represent barriers to implementation in the preceding seven days such as the facility headcount, the staffing complement, the availability of electricity and a functional computer, and how often the trained nurses had been allocated to implement eIMCI. After sufficient experience with obstacles to implementation, in-depth interviews with facility managers were conducted by a field worker (NN) in isiZulu according to a structured interview guide, were audio-recorded and subsequently translated into English and transcribed. Interview questions focused on how the facility managers allocated staff and how they identified staff for training and mentoring. The interview guide also included a brief survey to capture the allocations (current and previous) and the reasons for rotation of all facility staff present on the day of the interview.

Field notes and transcripts from the in-depth interviews were captured in Microsoft Excel 2016 and underwent manual content analysis. Quantitative elements from the database of eIMCI trained nurses, eIMCI use files, IT logs and quarterly site assessments were captured in Microsoft Excel 2016, and Excel data analysis tools were used for summary statistics, multilinear regressions and levels of significance. Associations were considered statistically significant at *p* < 0.05. The uptake and ongoing use of eIMCI were assessed by the following quarterly indicators: 1. The number of eIMCI records completed in the last seven days, and 2. The proportion of all child consultations undertaken by eIMCI during the last seven days. Barriers to use were identified by assessing the correlation of eIMCI use indicators with service platform variables during the last 7 days of each period, including the percentage of service time that electricity was available, a computer was available, the computer was functional, and the eIMCI trained nurse clinician was allocated to IMCI. Variables also included the under-5 daily workload per nurse clinician and the eIMCI training saturation. The eIMCI training saturation denotes the proportion of IMCI-trained nurses in the facility that were trained in eIMCI.

### Ethics

The study was approved by the uMgungundlovu Health Ethics Approval Board, the uMgungundlovu Health District Management and KwaZulu-Natal Department of Health.

## Results

### Characteristics of implementing facilities

Of the 53 primary healthcare facilities in uMgungundlovu District only 22 met the criteria for inclusion in the study. Of these all received ongoing implementation support but two were excluded in the analysis due to the lack of access to a computer during the entire study period. The two excluded facilities had participated in the 2018 pilot implementation but lost their computers in 2019 after a hardware fault and theft respectively, and the computers were not replaced by February 2021. Of the remaining 20 facilities (3 CHCs and 17 PHCs), 10 (50%) were situated in a rural, five (25%) in an urban and five (25%) in a peri-urban setting. Across data collection periods, the median (range) daily child under-5 headcount was 22 (7–50), the median child caseload per nurse was 19 (8–26) and the median number of facilities with adequate IT infrastructure for implementation was 18 (15–19). In November 2019, 30 nurse clinicians from the 22 facilities were capacitated in eIMCI with plans for 6-monthly workshops. However, the local COVID-19 outbreak prevented further workshops until November 2020 when 12 additional nurses were trained. Subsequent to training in eIMCI, 26/42 (62%) of nurse clinicians were allocated to work in the IMCI section and 24/42 (57%) gained sufficient experience to be certified in eIMCI. Characteristics of the nurse clinicians trained in eIMCI are displayed in Table 1.

**Table 1:** Characteristics of participants trained in eIMCI (*n* = 42)

Characteristic	Mean (range)
Age (years)	44 (29—62)
Duration of IMCI experience (years)	4 (0—17)
Pre-implementation computer literacy test score (%)	69 (0—100)
Improvement in computer literacy test scores during training (%)	32 (-6—97)
Pre-implementation IMCI knowledge test score (%)	54 (29—83)
Mean score during 4 skills assessments/mentor-observed consultations (%)	88 (65—98)
Mean duration of 4 mentor-observed consultations (minutes)	23 (10—45)

### The ongoing use of eIMCI

**Fig. 1:**
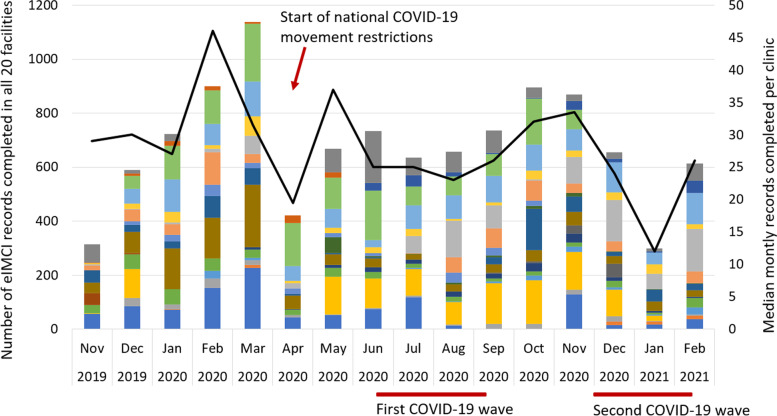
Monthly eIMCI uptake in uMgungundlovu district from Nov 2019—Feb 2021. The colours represent different facilities

During the study period from 1 November 2019 to 28 February 2021, 9 684 eIMCI records were completed across the 20 facilities in uMgungundlovu district, with a median uptake of 29 records per clinic per month. The local COVID-19 outbreak coincided with a decline in the monthly eIMCI uptake with national containment measures implemented from late March 2020 and epidemiological peaks occurring during the first wave from June—August 2020 and the second wave from December 2020 – January 2021 (Fig. 1).

**Fig. 2:**
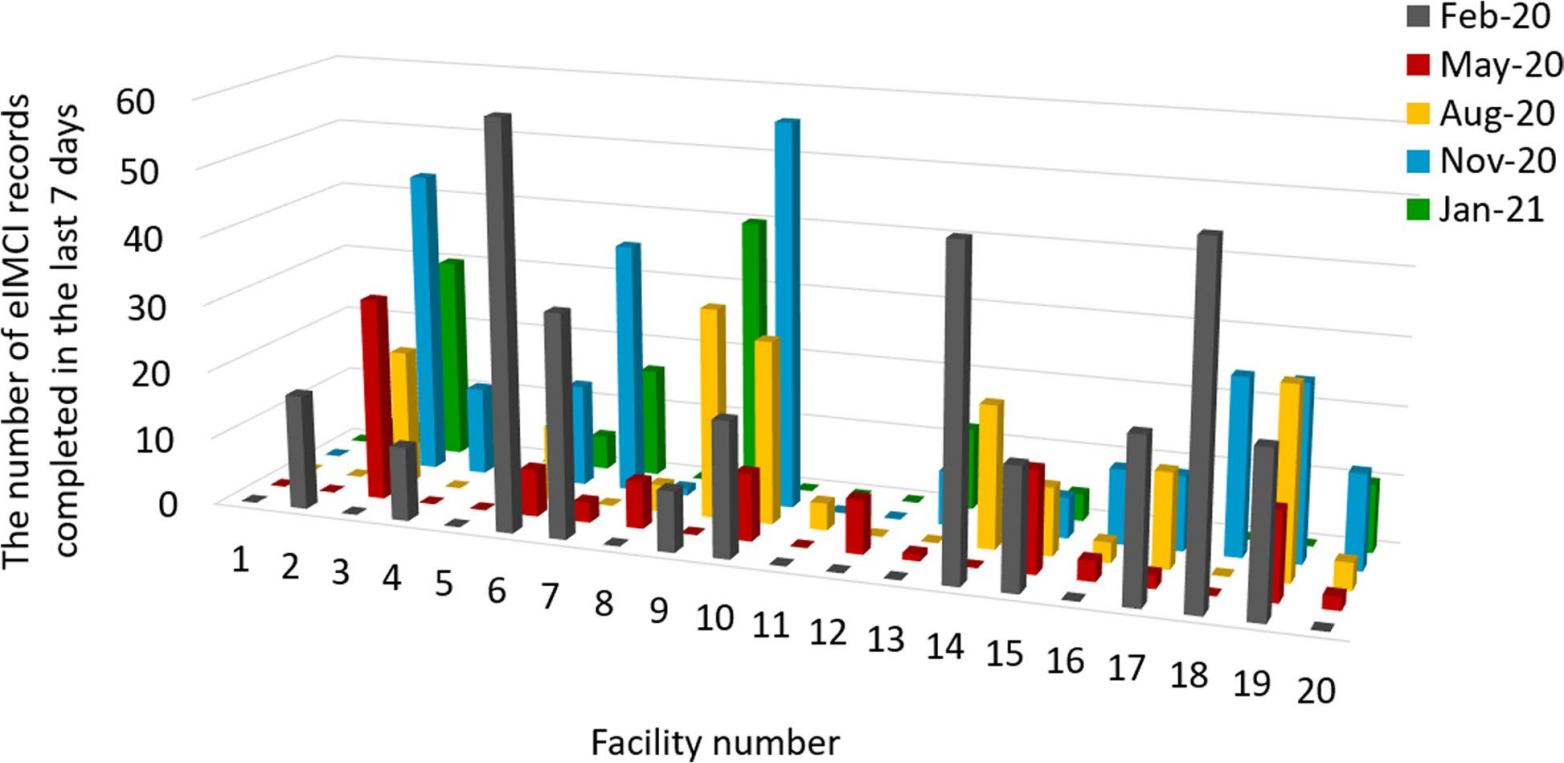
The number of eIMCI records completed in the last 7 days, quarterly by facility

**Fig. 3:**
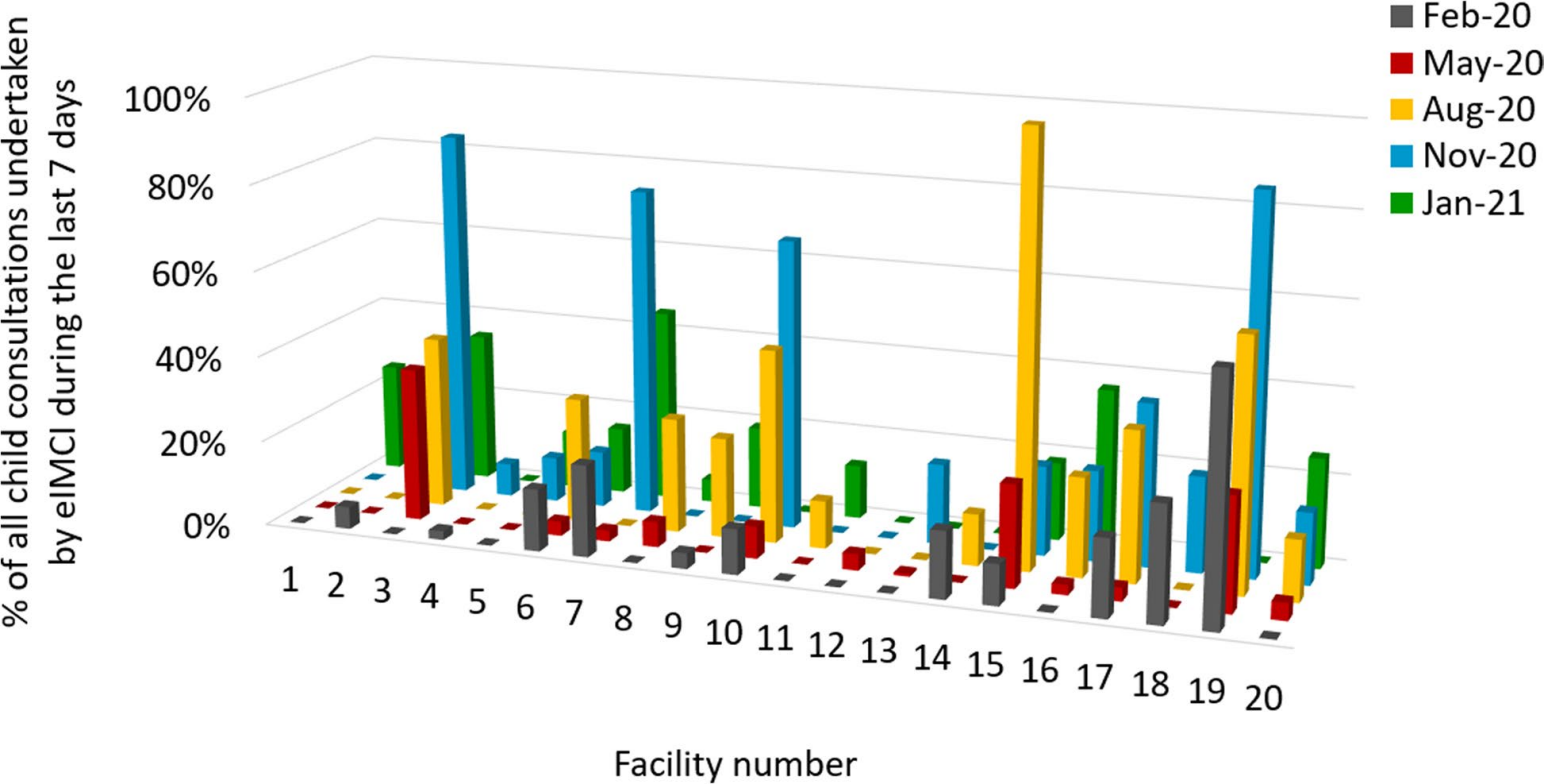
The proportion of all child consultations undertaken by eIMCI during the last seven days, quarterly by facility

Across 20 sites and five quarterly data collection periods, the mean (range) number of eIMCI records completed in the last seven days of each period was 10 (0–25) and the proportion of all child consultations undertaken by eIMCI 15% (1–46%). Substantial variation in use was observed between sites and within sites between data collection periods, as demonstrated in Figs. 2 and 3. In five facilities > 25% child consultations were undertaken by eIMCI (categorized as good use) and in seven facilities < 10% of child consultations were undertaken by eIMCI (categorized as poor use), with the remaining eight facilities falling between 10–25% (categorized as intermediate use).

### Barriers to eIMCI use

When the eIMCI use indicators were assessed for correlations to the service delivery platform indicators, significant associations were found only for the variables ‘service time last 7d eIMCI-trained nurse allocated’ (*p* < 0.001) and ‘daily child under-5 workload’ (*p* = 0.03), which together accounted for (R square) approximately 38% of the variability in ongoing eIMCI use. Table 2 displays the multivariate *p*-values for the use indicator ‘the number of eIMCI records completed in the last 7 days’. A corresponding regression for ‘the proportion of all child consultations undertaken by eIMCI during the last seven days’ demonstrated similar results.

**Table 2:** Factors associated with the number of eIMCI records completed in the last 7 days

**Regression Statistics**						
** Multiple R**	0.6179					
** R Square**	0.3818					
** Adjusted R Square**	0.3419					
** Standard Error**	11.4785					
** Observations**	100					
**ANOVA**	df	SS	MS	F	Significance F	
**Regression**	6	7567.44	1261.24	9.57	< 0.001	
**Residual**	93	12,253.30	131.76			
**Total**	99	19,820.75				
	Coefficients	Standard Error	t Stat	P-value	Lower 95%	Upper 95%
**Intercept**	10.7567	17.0004	0.6327	0.5285	-23.0026	44.5161
**% service time last 7 days eIMCI trained nurse available**	23.8693	3.7592	6.3495	< 0.001	16.4042	31.3344
**% service time last 7 days computer available**	2.0234	5.0501	0.4007	0.6896	-8.0052	12.0519
**% service time last 7 days functional computer**	2.8627	3.7174	0.7701	0.4432	-4.5194	10.2448
**% service time last 7 days electricity available**	-19.4261	15.7732	-1.2316	0.2212	-50.7486	11.8963
**eIMCI training saturation (%)**^**a**^	1.6060	5.9839	0.2684	0.7881	-10.2769	13.4889
**Daily child under-5 workload (n)**	0.3636	0.1677	2.1679	0.0327	0.0305	0.6967

^a^The number of nurses trained in electronic IMCI divided by the total number of IMCI trained nurses

The facilities with good eIMCI use differed from those with intermediate or poor use primarily with regards to the allocation of the eIMCI trained nurse (46% of the service time in the last seven days versus 32% and 12% respectively) and to a lesser degree with respect to the proportion of service time that the eIMCI computer was functional (Table 3).

**Table 3:** Quarterly eIMCI service platform and use indicators according to use category

eIMCI use category^a^	Good use	Intermediate use	Poor use
**Number of facilities**	5	8	7
**Service platform indicators**^**b**^			
Mean proportion of service time electricity was available	95%	95%	96%
Mean proportion of service time computer was available	92%	93%	89%
Mean proportion of service time the computer was functional (when available)	95%	93%	83%
Mean proportion of service time the trained nurse was allocated	46%	32%	12%
Mean daily child under-5 workload	16	19	17
**Use indicators**			
Mean number of eIMCI records completed	21	11	3
Mean proportion eIMCI of total child consultations	39%	16%	6%

^a^Use categories were based on the mean proportion of all child consultations undertaken by eIMCI during the last seven days across five data collection periods and were defined as ≥ 25% for facilities with good use, 10–25% for facilities with intermediate use and < 10% for facilities with poor use

^b^The service platform indicators were calculated as the mean for the last seven days during five data collection periods

Considerable temporal variation in eIMCI use was seen within facilities, with a median interquartile range (IQR) of 10% for ‘the proportion of all child consultations undertaken by eIMCI during the last seven days’ across different data collection periods. The majority (10/13) of the facilities with intermediate or good eIMCI use had a large variability between periods (IQR ≥ 10%). In these 10 facilities, the principal barriers during low-use periods included (percentage of facilities): staff shortages due to sick leave and COVID-19 related changes in the clinic function (40%), reallocation of the eIMCI trained nurse (30%), annual leave (20%), and resignation of the eIMCI trained nurse (10%). Computer challenges were a contributing barrier at three of the 10 facilities during low-use periods. The three facilities with more consistent use of eIMCI had similar challenges with staffing and computers, but not with reallocation or resignation of the eIMCI trained nurse. The chief implementation barriers in the seven facilities with consistently low use included (percentage of facilities): non-allocation of the eIMCI trained nurse (57%) and computer challenges (43%), with COVID-19-related staff shortages contributing at five facilities.

### Principal implementation barriers: staff allocation and computer challenges

The staff allocation and computer challenges warranted further analysis as they represented the most common barriers to ongoing eIMCI implementation in our context. During structured in-depth interviews, facility managers (*n* = 8) reported a median (range) staff rotation interval of 4.5 (3–12) months. The majority of managers (7/8 = 88%) listed staff skills and qualifications as their main criteria when determining allocation. Reasons given for rotating staff included the national Ideal Clinic Programme requirements for staff rotation and exposure to different clinical areas (5/8 = 63%), individual skills and ability to meet performance targets (2/8 = 25%) and staff exhaustion due to a high workload (1/8 = 13%). Three managers reported that nurses with specific skills and interests for a clinical area (champions) were infrequently rotated. When asked about how they identified participants for training workshops arranged by the health district, the majority (6/8 = 75%) of managers responded that they chose staff not previously trained in that area. Only one manager mentioned considering the selection criteria that the health district had set for the training. In contrast to the rotation interval reported by the facility managers, staff surveys (*n* = 40) revealed a median (range) allocation duration of 12 (1–336) months. Forty percent of the nurse clinicians surveyed were currently allocated to the maternal and child health stream and 53% reported that the last rotation had been requested by the facility manager. Nurse clinicians confirmed varying practices regarding allocation duration for staff within the same facility, stating that some had no fixed allocation (worked where it was needed) whereas champions with specific skills were rarely moved.

**Fig. 4:**
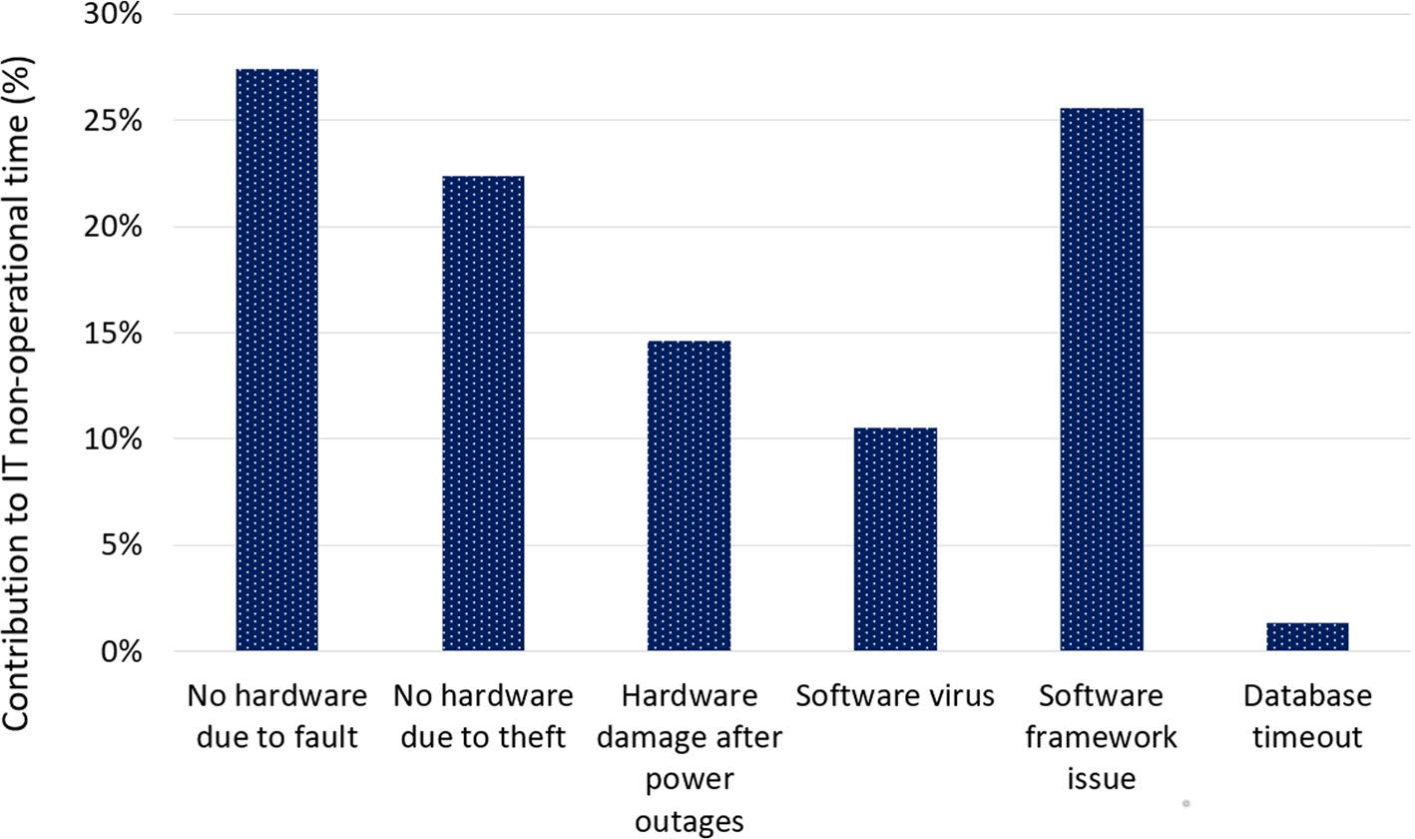
Hardware & software issues contribution to system downtime (% of total months downtime)

The computer challenges that posed the greatest barriers to eIMCI implementation are summarised in Fig. 4. Across the 20 facilities and 14-month study period, the median (range) number of non-operational months due to computer challenges was 0.6 (0.0–11.0) months, yielding a median (range) percentage computer downtime of 4.5% (0.0–78.6%). The computer downtime was < 2% in 10 facilities, 7% in one facility, 20–35% in 5 facilities and > 50% in 4 facilities. Hardware challenges accounted for 64% and software challenges for 36% of the overall non-operational time (Fig. 4). The commoner hardware issues contributing to the non-availability of the eIMCI computer were faults (27%) or theft (22%). Scheduled electricity interruptions (load-shedding) with subsequent power surges also resulted in irreparable hardware damage in two facilities. The software issues contributing most to the downtime were software framework issues (26%) and viruses (11%).

## Discussion

### Main study findings

During the 5 quarterly data collection periods, the majority of eIMCI facilities had a functional computer and a moderate caseload of children under 5 years. In keeping with what was seen during the pilot implementation, the ongoing use of eIMCI was modest and varied considerably between facilities and periods [16]. The uptake of electronic IMCI algorithms also waned over time in Tanzania where healthcare workers listed technical challenges, the length of consultations, drug supply, and staffing and workload problems as key barriers to their use [20]. A high staff turnover and difficulties in delivering supervision visits were challenges described in Burkina Faso [21]. In our setting, principal barriers to the ongoing use of eIMCI included non-allocation of eIMCI trained nurses, computer challenges and COVID-19-related disruptions in the service delivery. A higher caseload of children was positively associated with the use of eIMCI, i.e. represented a facilitator rather than a barrier to implementation. The reason for this finding is unclear, however, nurses with higher exposure to sick children may have developed better system familiarity and efficiency and/or may have a different appreciation for the complex nature of childhood illnesses which may influence their decision to use an electronic system that automates certain tasks and decisions. Unfortunately, much of the implementation period overlapped with the local COVID-19-outbreak, and a marked decline in the monthly eIMCI uptake was seen following the national movement restrictions and during epidemic peaks [22]. COVID-19-related health systems disruptions with deterioration in service access and service delivery have been duly documented elsewhere [23–25].

Previous studies evaluating IMCI implementation have highlighted a lack of support after didactic training and called for more comprehensive implementation models that also include mentoring, supervision and monitoring systems [1, 8–11]. With this in mind, the eIMCI capacitation model adopted a standardized approach to training, mentorship and supervision. However, despite clear training participant selection criteria and ongoing coordination with the facility managers, the post-training deployment of nurse clinicians was inferior even to that seen during the pilot implementation [16], with nearly 40% of nurses unable to practice eIMCI at their facility after the course. Sub-optimal allocation is a well-known barrier to building staff competence in our context, rendering mentoring and supervisory support difficult and necessitating continuous capacitation of new staff to sustain programme implementation [8, 11]. Interviews with facility managers confirmed little regard for the participant selection criteria given by training organisers and no uniform approach to staff allocation practices. The ability to meet performance targets for vertical programmes emerged as a key priority in facility managers’ allocation decisions. The conflicting priorities of various health programmes also contributed to district representatives being unavailable for collaboration and eIMCI capacitation and consequently rendered the DoH handover processes very difficult. Our study findings echo previously identified barriers to effective child health service delivery such as fragmented health systems with inadequate integration of vertical programmes; poorly defined roles and responsibilities; and a lack of officially adopted and institutionalized models for ongoing supervision and monitoring [1, 8–10].

Furthermore, few available computers at primary care facilities and the limited DoH capacity for IT support restricted the scale of eIMCI, with 31/53 (59%) of possible primary care facilities excluded because they had no computer for implementation. For implementing facilities, the resolution of computer challenges were heavily reliant on assistance from the dedicated eIMCI project team. Software-related challenges could more often be resolved, whereas many hardware challenges necessitated replacement of the equipment. The theft of computers proved a real challenge at eIMCI facilities. Stolen and broken computers were rarely replaced by the district DoH, which listed difficult procurement processes as an insurmountable barrier. Indeed, chronic procurement challenges have been lamented in South Africa and other low- and middle-income contexts [8, 9]. The scheduled electricity interruptions (load-shedding) did not directly represent a large proportion of the IT downtime during the implementation period. However, the subsequent power surges caused irreparable computer faults at two facilities, so that the overall impact was substantial.

### Study strengths and limitations

Study strengths included a long implementation period (14 months) which also enabled insights into programme sustainability. A reasonable sample size (20 facilities) and multiple data sources enabled a relatively comprehensive description of implementation challenges and health systems challenges. Limitations included a study period that overlapped with the local COVID-19 outbreak, likely skewing the feasibility results in a negative direction. The quarterly data collection periods did not allow for continuous day-to-day monitoring of the situation at the facilities, e.g. the staffing complement, allocation, electricity interruptions, computer challenges etc. Furthermore, when quantified, the identified implementation barriers could only account for a part of the variability in eIMCI use, and further qualitative work could have been useful to better shed light on various site-specific challenges. Nevertheless, we believe this study highlights important barriers to the ongoing implementation of electronic case management algorithms for children at a primary care level, and that similar challenges would apply to several other low- and middle-income contexts.

### Recommendations

Scaling up of eIMCI in South Africa would rely on resolving the identified local implementation challenges. Firstly, post-training allocation of staff would need to improve. Secondly, the capacity for IT support within the DoH would need a substantial lift. Thirdly, procurement processes to replace defective and missing equipment would need better definition and efficiency. Concurrent to the uMgungundlovu feasibility study, a study evaluating the effectiveness and cost-effectiveness of implementing the eIMCI model has been carried out in another KwaZulu-Natal district. Concluding whether or not overcoming the barriers to eIMCI implementation will be worth the investment will depend on the outcomes of this study.

## Conclusion

The ongoing eIMCI uptake was sporadic and the implementation undermined by barriers such as low post-training deployment of nurses; poor capacity in the DoH for IT support; and COVID-19-related disruptions in service delivery. Several challenges would need to be addressed for eIMCI implementation to be scaled in our context, including better adherence to criteria for participant selection and post-training deployment of staff; improved capacity in the DoH for IT support; more efficient procurement processes; and improved alignment of programmatic priorities.

## Data Availability

The datasets used and/or analysed during the current study are available from the corresponding author on reasonable request.

## Abbreviations

CHCs: Community health centres
DoH: Department of Health
eIMCI: Electronic IMCI
IT: Information technology
IMCI: Integrated Management of Childhood Illness
PHCs: Primary healthcare clinics
SA: South Africa

## Declarations

### Accordance

All methods were performed in accordance with relevant guidelines and regulations.
